# High‐Throughput Electron Diffraction Reveals a Hidden Novel Metal–Organic Framework for Electrocatalysis

**DOI:** 10.1002/anie.202016882

**Published:** 2021-04-07

**Authors:** Meng Ge, Yanzhi Wang, Francesco Carraro, Weibin Liang, Morteza Roostaeinia, Samira Siahrostami, Davide M. Proserpio, Christian Doonan, Paolo Falcaro, Haoquan Zheng, Xiaodong Zou, Zhehao Huang

**Affiliations:** ^1^ Department of Materials and Environmental Chemistry Stockholm University 10691 Stockholm Sweden; ^2^ Key Laboratory of Applied Surface and Colloid Chemistry Ministry of Education School of Chemistry and Chemical Engineering Shaanxi Normal University Xi'an 710119 China; ^3^ Institute of Physical and Theoretical Chemistry Graz University of Technology Stremayrgasse 9 8010 Graz Austria; ^4^ Department of Chemistry and the Centre for Advanced Nanomaterials The University of Adelaide Adelaide 5005 South Australia Australia; ^5^ Department of Chemistry University of Calgary 2500 University Drive NW Calgary Alberta T2N1N4 Canada; ^6^ Dipartimento di Chimica Università degli Studi di Milano 20133 Milano Italy; ^7^ Samara Center for Theoretical Materials Science (SCTMS) Samara State Technical University Samara 443100 Russia

**Keywords:** continuous rotation electron diffraction, electrocatalysis, high throughput structural analysis, metal–organic frameworks, three-dimensional electron diffraction

## Abstract

Metal‐organic frameworks (MOFs) are known for their versatile combination of inorganic building units and organic linkers, which offers immense opportunities in a wide range of applications. However, many MOFs are typically synthesized as multiphasic polycrystalline powders, which are challenging for studies by X‐ray diffraction. Therefore, developing new structural characterization techniques is highly desired in order to accelerate discoveries of new materials. Here, we report a high‐throughput approach for structural analysis of MOF nano‐ and sub‐microcrystals by three‐dimensional electron diffraction (3DED). A new zeolitic‐imidazolate framework (ZIF), denoted ZIF‐EC1, was first discovered in a trace amount during the study of a known ZIF‐CO_3_‐1 material by 3DED. The structures of both ZIFs were solved and refined using 3DED data. ZIF‐EC1 has a dense 3D framework structure, which is built by linking mono‐ and bi‐nuclear Zn clusters and 2‐methylimidazolates (mIm^−^). With a composition of Zn_3_(mIm)_5_(OH), ZIF‐EC1 exhibits high N and Zn densities. We show that the N‐doped carbon material derived from ZIF‐EC1 is a promising electrocatalyst for oxygen reduction reaction (ORR). The discovery of this new MOF and its conversion to an efficient electrocatalyst highlights the power of 3DED in developing new materials and their applications.

## Graphical abstract

Three‐dimensional electron diffraction can be used as a high‐throughput technique for discovering new materials, such as metal–organic frameworks, especially when they are synthesized in a phase mixture.

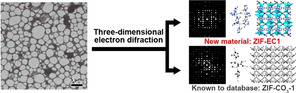

## Introduction

Metal‐organic frameworks (MOFs), or porous coordination polymers (PCPs), are a class of highly crystalline and porous hybrid materials constructed by linking metal clusters (or ions) and organic ligands via coordination bonds.[^1^, ^2^] In addition, their tunable structure metrics and topologies give rise to versatile properties,^[3]^ and vast opportunities for applications in gas storage,[^4^, ^5^] separation,[^6^, ^7^, ^8^, ^9^] catalysis,[^10^, ^11^, ^12^] energy conversion and storage,[^13^, ^14^, ^15^, ^16^, ^17^, ^18^, ^19^] and bio‐medical science.[^20^, ^21^] With the access to almost unlimited combinations of inorganic building units and organic linkers, more than 80 000 different MOFs have been reported over the past two decades.^[22]^ Interestingly, through the control of reaction kinetics or thermodynamics, different structures with distinct properties can be obtained even using the same building units.[^23^, ^24^, ^25^, ^26^] A relevant example is the large sub‐class of MOFs termed zeolitic imidazolate frameworks (ZIFs),^[27]^ which are synthesized by connecting tetrahedrally‐coordinated metal ions and linkers. These components can lead to a variety of topologies such as **sod**,[^28^, ^29^, ^30^] **crb**,^[29]^ **dia**,^[30]^ **poz**,^[31]^ etc. Consequently, ZIFs with different topologies can commonly coexist in the bulk polycrystalline product. Accompanied by the tiny crystal sizes which are inaccessible to single crystal X‐ray diffraction (SCXRD), structural characterization of these materials poses a major challenge, particularly when in search for new materials.

Powder X‐ray diffraction (PXRD) is the most widely used technique for characterization of polycrystalline materials. However, PXRD has a major drawback of peak overlapping, hindering accurate peak indexing and intensity extraction. This becomes more severe for samples containing several phases, which leads to either wrong phase assignments or no solution at all. Phase mixtures or polymorphs are often produced during the development of new materials. The aforementioned drawback of PXRD makes it especially challenging to study multiphasic materials containing new phases, which are likely to be overlooked, therefore preventing the discovery of new materials. Furthermore, the peak overlapping makes ab initio structure determination difficult and, in many cases, impossible. For MOFs, structural studies are even more difficult due to their relatively large unit cells that intensify the drawback of peak overlapping in PXRD patterns. These challenges are well tackled with the recent development of three‐dimensional electron diffraction (3DED).[^32^, ^33^, ^34^] Benefited from the strong interaction between electrons and matter, 3DED allows single crystal structural analysis even when the crystal sizes are down to the range of nanometers.[^35^, ^36^, ^37^, ^38^, ^39^] This turns a polycrystalline powder into millions of analytes of single crystals. With a short data collection time of 3–5 minutes per crystal, it is therefore possible to analyze individual crystals in a high throughput manner and determine the structure of each tiny crystal in a phase mixture. More importantly, new materials in trace amounts can be discovered and their structures imparting unique properties can be revealed by 3DED.

Here, we report the first use of a 3DED technique, continuous rotation electron diffraction (cRED), in discovery of a new MOF among a phase mixture. The new MOF, denoted as ZIF‐EC1 (EC: structure solved by **E**lectron **C**rystallography), is constructed by linking Zn^II^ cations and deprotonated 2‐methylimidazole (mIm^−^) linkers. It was discovered by cRED with a trace amount in a ZIF‐CO_3_‐1 material. The atomic structures of both MOFs were successfully determined by cRED. Interestingly, the structure of ZIF‐EC1 is rather dense, which is built by mono‐ and binuclear Zn clusters. This offers a high density of N and Zn, which are active sites for electrocatalysis.[^40^, ^41^] Density functional theory (DFT) calculations show ZIF‐EC1 has a higher stability than ZIF‐CO_3_‐1. This provided insights for successfully obtaining a phase pure ZIF‐EC1 material, which is important for catalysis. We subsequently converted pure ZIF‐EC1 to N‐doped carbon material as an electrocatalyst for oxygen reduction reaction (ORR). Due to the highly dense structure of ZIF‐EC1, it leads to high contents of N and Zn in the derived carbon material. In addition, the oxygen atoms in the framework of ZIF‐EC1 assist to generate a porous structure with high surface area, which is favorable for mass transfer. Owing to these advantages, the carbon material derived from ZIF‐EC1 exhibits the best performance compared to those derived from other ZIFs including ZIF‐1, ZIF‐8, and ZIF‐95. Our strategy by using cRED as a high throughput analytical tool would benefit communities beyond the MOF field to accelerate research in developing new materials.

## Results and Discussion

**Figure 1:**
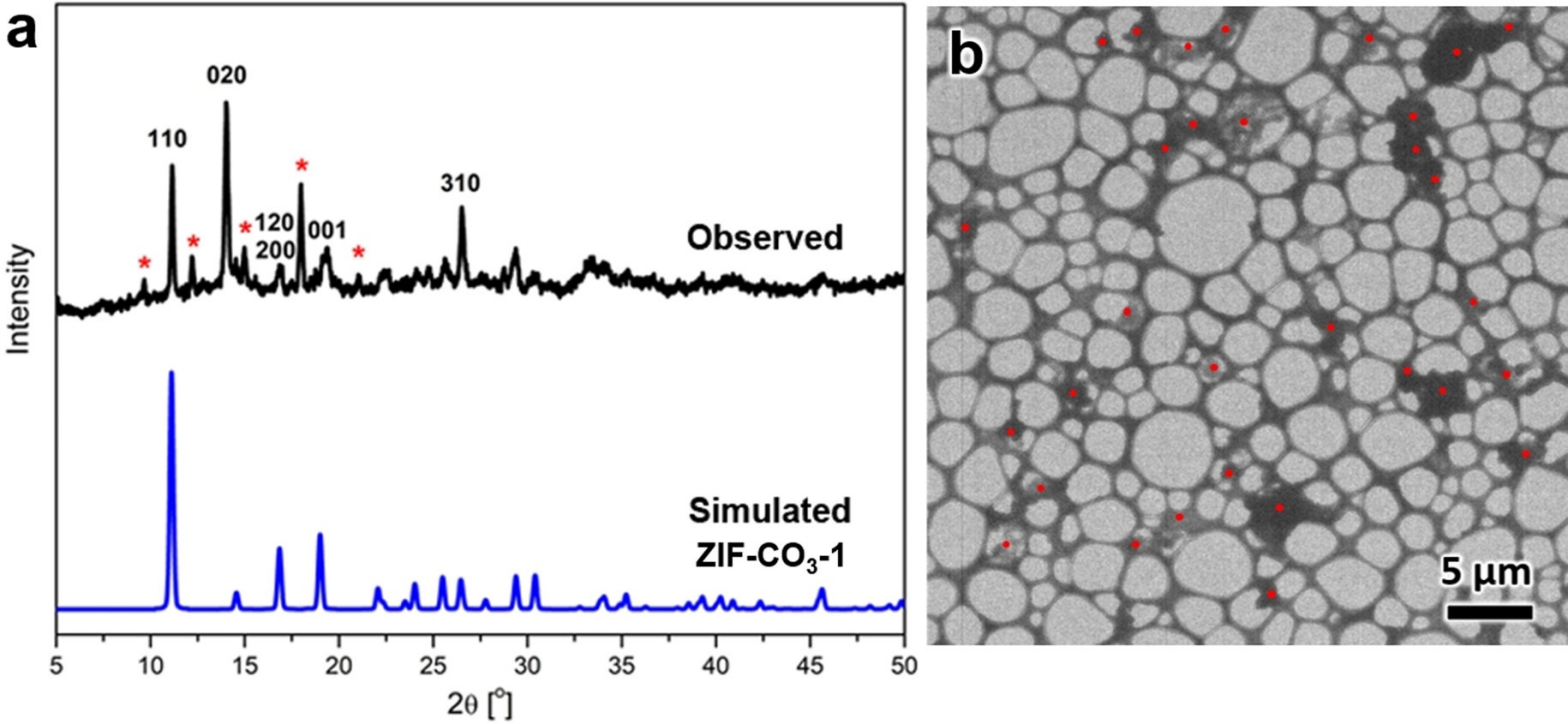
a) Comparison of observed PXRD pattern (*λ*=1.5406 Å) with simulated pattern from the structural model of ZIF‐CO_3_‐1. Many strong peaks (marked by asterisks) remain unidentified. b) TEM image showing individual nanocrystals (marked by red dots) in an area of 35×35 μm^2^ studied by cRED.

PXRD is widely used to analyze polycrystalline products. Typically, structural analysis is done by matching peak positions in the experimental PXRD pattern with those calculated from possible structures in a crystallographic database, for example, the Cambridge Structure Database, which includes more than one million reported crystal structures.^[22]^ Nevertheless, this is often very challenging as shown in the case of the new ZIF‐EC1 discovery. Using Zn^II^ cations and mIm^−^ as the organic linker, we obtained a polycrystalline product, which shows a variation of particle sizes (0.2–5 μm) and morphologies (Figure S1). The PXRD pattern presents strong and sharp peaks, which indicates the sample has a good crystallinity (Figure 1 a). However, it was difficult to index the PXRD pattern, and the material was initially regarded as a pure new phase (denoted as U14).^[42]^ Only after our investigations of ZIF‐CO_3_‐1 by cRED,^[34]^ we could identify ZIF‐CO_3_‐1 as the major phase in the sample (Figure 1 a). Yet, there are many peaks in the PXRD pattern that cannot be identified. As in most phase mixtures, the number of unindexed peaks belonging to a minor phase is too few that prevents phase identification and new structure determination.

**Figure 2:**
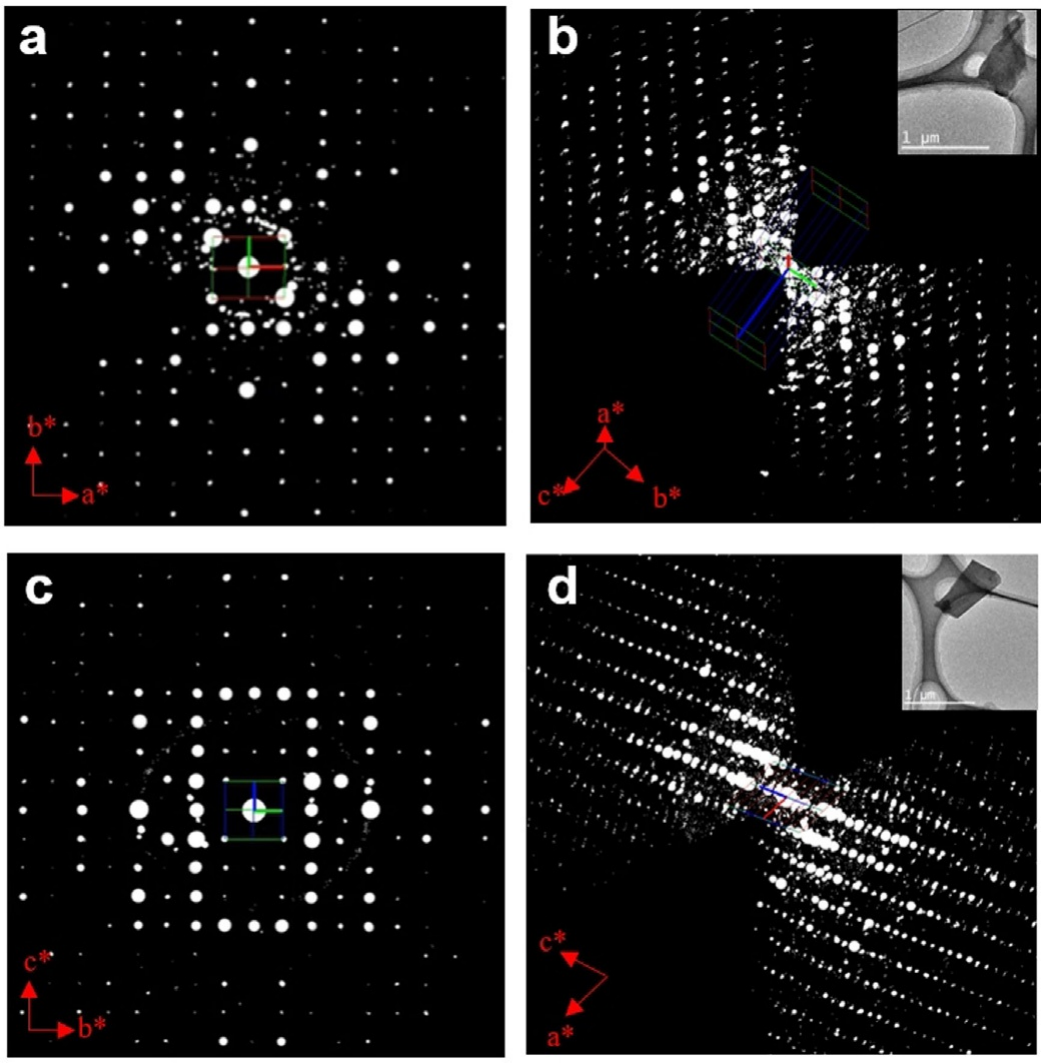
Reciprocal lattices reconstructed from cRED data. a,b) 2D slice showing the *hk*0 plane (a) cut from the 3D reciprocal lattice (b) of a ZIF‐CO_3_‐1 crystal shown as the inset in (b). c,d) 2D slice showing the 0*kl* plane (c) cut from the 3D reciprocal lattice (d) of a ZIF‐EC1 crystal shown as the inset in (d). Apart from the Bragg reflections, weak spots are attributed to the background noise. In addition, the amorphous carbon from the TEM grid and the bovine serum albumin (BSA) molecules used in the synthesis could also generate weak spots.

3DED was applied to uniquely tackle these challenges on structural analysis of the new phase. It allows to identify and collect data from single nano‐ and sub‐microcrystals. Remarkably, with the recent evolution of 3DED methods,^[43]^ data collection time has been reduced to a few minutes per crystal, providing a new strategy for high throughput phase analysis and crystal structure determination. As shown in the TEM image in Figure 1 b, more than 30 particles can be found and analyzed in an area of 35×35 μm^2^. cRED data were collected from 11 individual nano‐ and sub‐microcrystals with the rotation angles ranging from 39.26 to 117.45°, and the total data collection time of 1.5 to 4.3 min (Table S1, see Supporting Information for more details). By analyzing 3D reciprocal lattices reconstructed from the 11 cRED datasets, two distinct crystal systems and unit cells are revealed (Figure 2 and Figure S2). Nine crystals have an orthorhombic unit cell, with *a*=10.50 Å, *b*=12.51 Å, and *c*=4.69 Å and the remaining two exhibit a monoclinic unit cell, with *a*=13.58 Å, *b*=14.55 Å, *c*=14.31 Å, and *β*=118.0°. The space group was deduced from the reflection conditions observed from the reconstructed reciprocal lattice, which is *Pba*2 (No. 32) or *Pbam* (No. 55) for the former nine crystals and *P*2_1_/*c* (No. 14) for the latter two. The unit cell parameters and space group of the orthorhombic crystals agree to those of the ZIF‐CO_3_‐1 phase as previously determined from pure samples.[^34^, ^44^] Meanwhile, no reported ZIFs match the unit cell and space group for the second phase, indicating it is a new MOF, which we denoted as ZIF‐EC1.

**Figure 3:**
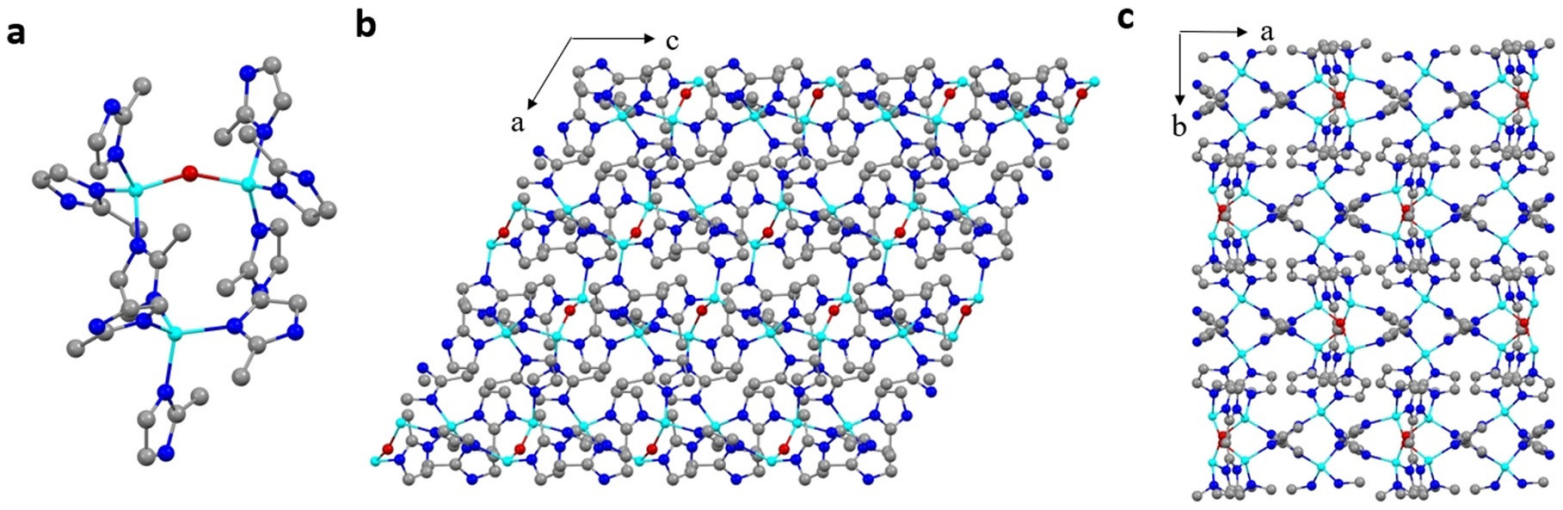
Structural model of ZIF‐EC1. a) The coordination geometry of Zn. b,c) The framework structure viewing along *b*‐ and *c*‐axis, respectively. Cyan spheres: Zn atoms; red spheres: O atoms; blue spheres: N atoms; grey spheres: C atoms. H atoms are not shown.

Using the SHELX software package,^[45]^ ab initio structure determination was applied on each of the cRED datasets. The positions of all non‐hydrogen atoms were found directly from the structure solution by direct methods. For the ZIF‐CO_3_‐1 phase, the obtained structure is consistent with that determined by SCXRD (Figure S3). For the trace amount of the new ZIF‐EC1 in the phase mixture, all three Zn^II^ cations and five mIm^−^ linkers in the asymmetric unit were located and the non‐hydrogen atoms were refined anisotropically (see Table S2 for more details). ZIF‐EC1 has a general formula of Zn_3_(mIm)_5_(OH). Each mIm^−^ linker connects to two Zn atoms. One of the three Zn cations is connected to four mIm^−^ linkers to form a ZnN_4_ mononuclear cluster while the other two are coordinated to three mIm^−^ linkers and one bridging OH^−^ group to form a binuclear Zn_2_N_6_(OH) cluster (Figure 3 a). ZIF‐EC1 is nonporous as shown in Figures 3 b and c and S4. Topological analysis (Figure S5) of the ZIF‐EC1 framework using ToposPro^[46]^ shows a rarely reported *yqt*1 topology[^47^, ^48^, ^49^] as found in the Samara Topological Data Center.^[50]^ PXRD patterns simulated from the structural models of ZIF‐CO_3_‐1 and ZIF‐EC1 are compared to the experimental PXRD pattern (Figure S6). All the peaks in the experimental PXRD pattern can finally be indexed by these two phases.

**Figure 4:**
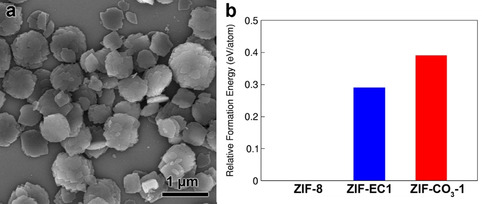
a) SEM image of pure ZIF‐EC1 nanocrystals. b) Calculated relative formation energies using DFT calculations. The formation energies are calculated per atom for each ZIF structure. The formation energy of ZIF‐8 is set to zero and the formation energy of ZIF‐EC1 and ZIF‐CO_3_‐1 are calculated relative to ZIF‐8.

Despite the absence of porosity, ZIF‐EC1 based on Zn mono‐ and binuclear clusters provides a higher density of metal and N sites as compared to ZIFs with only mononuclear Zn clusters (Table 1). The high density is favorable for catalysis. However, in order to study the properties and catalytic performance, a pure ZIF‐EC1 material is highly desired. We therefore applied density functional theory (DFT) calculation to obtain the formation energy per atom and thus the stability of ZIF‐CO_3_‐1 and ZIF‐EC1. We used ZIF‐8 as the reference since it has the lowest energy per atom (Figure 4 b). These results indicate that ZIF‐EC1 is thermodynamically more stable than ZIF‐CO_3_‐1.

**Table 1:** Comparison of metal and nitrogen density of ZIF‐EC1 to most reported ZIFs.

Name	Net	Composition	Density (Zn atoms nm^−3^)^[a]^	Density (N atoms nm^−3^)^[a]^	Reference
**ZIF‐EC1**	*yqt*1	**Zn_3_(mIm)_5_(OH)**	**4.77**	**15.90**	**This work**
ZIF‐1	**crb**	Zn(Im)_2_	3.64	14.56	[29]
ZIF‐2	**crb**	Zn(Im)_2_	2.80	11.20	[29]
ZIF‐3	**dft**	Zn(Im)_2_	2.66	10.64	[29]
ZIF‐4	**cag**	Zn(Im)_2_	3.68	14.72	[29]
ZIF‐6	**gls**	Zn(Im)_2_	2.31	9.24	[29]
ZIF‐8	**sod**	Zn(mIm)_2_	2.47	9.88	[28, 29]
ZIF‐10	**mer**	Zn(Im)_2_	2.25	9.00	[30]
ZIF‐14	**ana**	Zn(eIm)_2_	2.57	10.28	[30, 51]
ZIF‐22	**lta**	Zn(5abIm)_2_	2.02	8.08	[52]
ZIF‐23	**dia**	Zn(4abIm)_2_	3.32	13.28	[52]
ZIF‐71	**rho**	Zn(dcIm)_2_	2.06	8.24	[30]
ZIF‐77	**frl**	Zn(nIm)	3.22	12.88	[30]
ZIF‐95	**poz**	Zn(cbIm)_2_	1.51	6.04	[31]
ZIF‐100	**moz**	Zn_20_(mIm)_39_(OH)	1.29	5.03	[31]

[a] See the Supporting Information for more details on calculating the density. Im=imidazolate; eIm=2‐ethylimidazolate; 5abIm=5‐azabenzimidazolate; 4abIm=4‐azabenzimidazolate; dcIm=4,5‐dicnoroimidazolate; nIm=2‐nitroimidazolate; cbIm=5‐chlorobenzimidazolate.

Nucleation rate plays an important role in MOF syntheses. Typically, the introduction of a competing reagent or modulator during the synthesis can inhibit and slow down the nucleation, which facilitates the formation of metastable crystalline products.^[53]^ Given that Zn(OAc)_2_⋅2 H_2_O could react rapidly with HmIm to generate crystals at room temperature, the nucleation kinetics is crucial. To inhibit the formation of the metastable phase ZIF‐CO_3_‐1 and promote the formation of the thermodynamically stable phase of ZIF‐EC1, we used a synthetic condition that favors the nucleation.^[54]^ Besides using an excess amount of HmIm (molar ratio HmIm/Zn=16), the reaction of Zn(OAc)_2_⋅2 (H_2_O) and HmIm was conducted under vigorous stirring to enhance the nucleation rate. Under this condition, a pure ZIF‐EC1 sample was obtained. SEM images show that crystals of pure ZIF‐EC1 have plate‐like morphology (Figure 4 a). Pawley fitting was applied to confirm the purity of the sample, which shows a good agreement between the observed and calculated patterns (Figure S7 and Table S3). In addition, ab initio structure determination was performed on the pure ZIF‐EC1 phase, and the same crystal structure was obtained as that in the phase mixture (Figures S8, S9 and Table S2).

**Figure 5:**
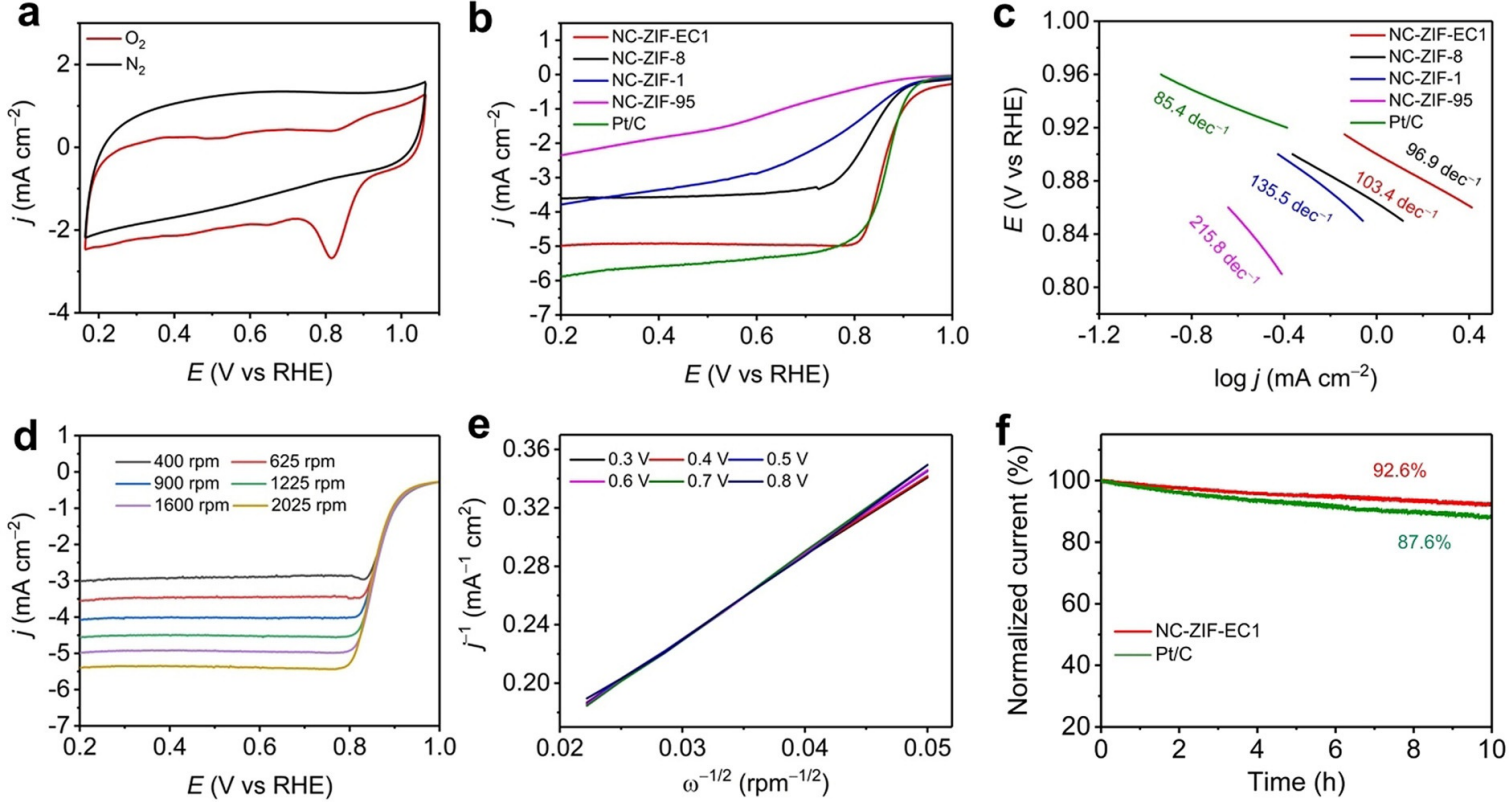
a) CV curves of NC‐ZIF‐EC1 in 0.1 M KOH solution saturated by N_2_ and O_2_, respectively. b) LSV curves and c) Tafel plots of NC‐ZIF‐EC1, NC‐ZIF‐1, NC‐ZIF‐8, NC‐ZIF‐95, and Pt/C. d) LSV curves of NC‐ZIF‐EC1 at different rotating speeds. e) *K*–*L* plots at different potentials derived from data in (d). f) *j*–*t* chronoamperometric responses at 0.664 V (vs. RHE).

3DED provides a new strategy in searching for novel materials. Meanwhile, the structural insights obtained by 3DED reveal that the binuclear‐based ZIF‐EC1 could enhance catalytical performance through its high density of N and Zn. We therefore demonstrate ZIF‐EC1 and its N‐doped carbon derivative as electrocatalysts for ORR (Figures 5 and S10). SEM image shows that the morphology of nitrogen‐doped carbon (NC) derived from ZIF‐EC1 (denoted as NC‐ZIF‐EC1) after pyrolysis at 900 °C in Ar atmosphere remained the same as that of the pristine ZIF‐EC1 (Figure S11). Applied as an electrocatalyst, NC‐ZIF‐EC1 exhibits an obvious oxygen reduction peak appearing at near 0.85 V in the cyclic voltammetry (CV) curve, confirming its electrocatalytic oxygen reduction activity (Figure 5 a). The linear sweep voltammetry (LSV) shows that NC‐ZIF‐EC1 achieved promising onset potential (*E*_onset_=0.930 V) and half‐wave potential (*E*_1/2_=0.860 V), which is comparable to that of Pt/C (*E*_1/2_=0.867, Figure 5 b). Due to the Zn binuclear cluster, ZIF‐EC1 has a high density of N and Zn, which is 1.3–3.2 times higher than those in most reported low density ZIFs, such as ZIF‐1, ZIF‐8, and ZIF‐95. Consequently, the weight percentages of N and Zn atoms, which serve as the active sites, are 2.1–4.3 times higher in NC‐ZIF‐EC1 than those in the nitrogen‐doped carbons derived from ZIF‐1, ZIF‐8, and ZIF‐95 (denoted as NC‐ZIF‐1, NC‐ZIF‐8, and NC‐ZIF‐95, respectively, Figures S12, S13, and Table S4). As a result, NC‐ZIF‐EC1 exhibits a better ORR activity than do the NC‐ZIF‐1, NC‐ZIF‐8, and NC‐ZIF‐95 (Figures 5 b, S14–S17 and Table S5). In addition, ZIF‐EC1 contains O atoms in the framework structure, which partially removes C atoms by evolution of CO/CO_2_ during the pyrolysis.[^55^, ^56^] This not only reduces the carbon proportion in the NC‐ZIF‐EC1, but also facilitate the generation of large pores and high surface area (Figure S18), which contribute to the ORR activity by creating favorable mass transfer pathways. PXRD patterns of NCs show that all materials have partially graphitized structures (Figure S19a), while Raman spectra exhibit a similar amount of defective structure, indicating a similar graphitized structure among the NC materials (Figure S19b). By taking these advantages, in addition, NC‐ZIF‐EC1 outperforms most of the other state‐of‐the‐art electrocatalysts, including MOFs,[^57^, ^58^] COFs,[^59^, ^60^] MOF derived carbon materials,[^61^, ^62^, ^63^, ^64^] carbon materials,[^41^, ^65^, ^66^, ^67^] and metal oxides,[^68^, ^69^] etc. (Table S6).

The electron transfer number (*n*) of NC‐ZIF‐EC1 was calculated to be ≈4.0 based on *K*–*L* plots, and ≈3.6 based on rotating ring disk electrode (RRDE) measurement (Figures 5 d, e, and S20, see Supporting Information for more details). This indicates an outstanding 4 e^−^ selectivity, which is a favorable reaction pathway to provide high reaction kinetics and prolong the durability of the cell. Combined with a higher density, which favors charge transfer during the electrocatalysis, NC‐ZIF‐EC1 shows a better reaction kinetics as indicated by the lower Tafel slope compared to than those in NC‐ZIF‐1, NC‐ZIF‐8, and NC‐ZIF‐95 (Figure 5 c). The long‐term durability of NC‐ZIF‐EC1 was evaluated using *j*–*t* chronoamperometric responses (Figure 5 f). After 10 h, the current density of NC‐ZIF‐EC1 only dropped by 7.4 %, which suggests a better stability compared to that of Pt/C. In addition, post‐catalysis characterization shows that the morphology of NC‐ZIF‐EC1 remains intact after the ORR reactions (Figure S21).

## Conclusion

By applying high throughput structural analysis on single nanocrystals of a phase mixture, we discovered a new MOF, ZIF‐EC1. By controlling the reaction kinetics, pure ZIF‐EC1 was synthesized. We present a proof‐of‐concept study that highlights the advantage of the 3DED technique in the development of MOF materials. Thanks to the new strategy, phases in trace amount and tiny crystals can be studied, which could accelerate the search for new MOFs. Revealing the structure uncovers the key property of ZIF‐EC1, as it contains high density of metal sites and N atoms. As a result, the carbon material derived from ZIF‐EC1 shows excellent electroactivity as compared to those of other Zn^II^ cation based ZIFs. We foresee that the application of 3DED technique will have a broad impact not only in the development of MOFs, but also in a wide range of materials and chemical compounds, such as metal oxides, polyoxometalates, and pharmaceutical compounds, where pure phases and large single crystals are difficult to obtain.

Deposition Number 2046826 contains the supplementary crystallographic data for this paper. These data are provided free of charge by the joint Cambridge Crystallographic Data Centre and Fachinformationszentrum Karlsruhe Access Structures service www.ccdc.cam.ac.uk/structures.

## Conflict of interest

The authors declare no conflict of interest.

## Supporting information

As a service to our authors and readers, this journal provides supporting information supplied by the authors. Such materials are peer reviewed and may be re‐organized for online delivery, but are not copy‐edited or typeset. Technical support issues arising from supporting information (other than missing files) should be addressed to the authors.

Supplementary
