# Supplementary material for: High‐Throughput Electron Diffraction Reveals a Hidden Novel Metal–Organic Framework for Electrocatalysis
Source: Angew Chem Int Ed Engl. 2021 Apr 7;60(20):11391–7. doi: 10.1002/anie.202016882 (PMC8252586; doi:10.1002/anie.202016882)
Supplement: Supplementary file 1 — Supplementary [file ANIE-60-11391-s001.pdf]

Angewandte Chemie
Eine Zeitschrift der Gesellschaft Deutscher Chemiker

Supporting Information

High-Throughput Electron Diffraction Reveals a Hidden Novel Metal–Organic Framework for Electrocatalysis

Meng Ge+, Yanzhi Wang+, Francesco Carraro, Weibin Liang, Morteza Roostaeinia, Samira Siahrostami, Davide M. Proserpio, Christian Doonan, Paolo Falcaro, Haoquan Zheng, Xiaodong Zou, and Zhehao Huang**

anie_202016882_sm_miscellaneous_information.pdf

Table of Contents:

Section 1. Materials and instrumentation

Section 2. Synthesis of ZIF-EC1

Section 3. Structural analysis by cRED

Section 4. Energy calculation of ZIF-EC1

Section 5. Electrochemical analysis of ZIF-EC1 and its derivatives

Section 1. Materials and instrumentation

Materials.

$Zn(NO_3)_2 \cdot 6H_2O$ (99.9%), $Zn(OAc)_2 \cdot 2H_2O$ (99.9%), 2-methylimidazole (HmIm) (99%), Imidazole (HIm) (99%), 5-chlorobenzimidazole (HcbIm) (97%) were purchased from the Energy Chemical. Ethanol (AR, 99.5%), Methanol (AR, 99.5%), N,N-Dimethylformamide (AR, 99.5%), Dichloromethane (AR, 99.5%) were purchased from Sinopharm Chemical Reagent Co. All the reagents were used without further purification unless otherwise mentioned. Deionized water was used throughout the experimental processes.

Methods.

Scanning electron microscopy (SEM). SEM images were performed on a JEOL JSM-7000F scanning electron microscope with a field emission gun capable of generating and collecting high-resolution and low-vacuum images. The field emission gun assembles with a Schottky emitter source. The images were obtained at an accelerating voltage of 15.0 kV, with beam current of 200 μA.

Powder X-ray diffraction (PXRD). PXRD patterns were recorded on a PANalytical X'Pert Pro diffractometer equipped with a Pixel detector and a monochromator, using Cu $K_{\alpha 1}$ radiation (λ = 1.5406 Å). Data was recorded using a current of 40 mA, accelerating voltage of 40 V and source silt 15 mm.

Electrochemical evaluation. All electrochemical tests were carried out on CHI 660E electrochemical workstation at 30 °C. Oxygen reduction reaction (ORR) performances were evaluated by a three-electrode system. The working electrode was a rotating disk electrode (RDE) with a diameter of 5 mm (0.196 cm^2). The counter electrode was a graphite rod and the reference electrode was a saturated Ag/AgCl electrode. The cyclic voltammetry (CV) test was carried out in 0.1 M KOH saturated with O_2 (scan rate is 50 mV s^{-1}). The linear sweep voltammetry (LSV) test was carried out in 0.1 M KOH saturated with O_2, and the speed range was 400 to 2025 rpm (scan rate was 5 mV s^{-1}). Current-time chronoamperometric responses were measured at 0.66 V (relative to RHE). The catalyst ink for ORR tests was prepared as the following: 4 mg catalyst was dispersed in a mixed solution containing 660 μL isopropanol, 330 μL deionized water and 10 μL Nafion aqueous solution (5 wt%). The mixed solution was treated by ultrasound for 1 h to form a homogeneous suspension. Then 20 μL catalyst solution was taken from the pipette and uniformly dripped onto the rotating disc electrode (RDE) (catalyst loading was about 0.4 mg cm^{-2}). After natural drying, a homogeneous film was formed.

Section 2. Synthesis of ZIF-EC1

Synthesis of ZIF-EC1 and ZIF-CO_3-1 mixture. In a typical synthesis, 300 μL of a 440 mM aqueous solution of HmIM, 60 μL of a 36 mg mL^{-1} aqueous solution of bovine serum albumin (BSA) and 1140 μL of deionized water were mixed in a 2 ml plastic centrifuge tube for 1 minute. Then, the solution was added to 500 μL of an 80 mM aqueous solution of $Zn(OAc)_2 \cdot 2(H_2O)$. The

mixture was left under static conditions at RT for 24 h. White powder was then harvested by centrifugation followed by washing by deionized water for at least six times.

Synthesis of pure ZIF-EC1. In a typical synthesis of pure ZIF-EC1, 0.125 mL of a 3.84 M aqueous solution of HmIM was mixed with 1.875 mL of deionized water. 1 mL of 0.24 M aqueous solution of $Zn(OAc)_2·2(H_2O)$ was added into the above solution under vigorous stirring condition. The mixture was kept under stirring at RT for at least 4 h. White powder was then harvested by centrifugation followed by washing by deionized water for at least six times.

Synthesis of ZIF-8. In a typical synthesis of pure ZIF-8, 100 mg HmIm was dissolved in 10 mL of methanol to form solution A. 140 mg $Zn(NO_3)_2·6H_2O$ was dissolved in another 10 mL methanol to form solution B. Then, solution B was poured into solution A under magnetic stirring for 10 min and aged for 24 h at room temperature. The product was collected by centrifugation and washed with methanol. It was then dried overnight in a freeze dryer for characterization.

Synthesis of ZIF-1. In a typical synthesis of pure ZIF-1, 100 mg $Zn(NO_3)_2·6H_2O$ and 150 mg HIm are dissolved in a glass bottle containing 18 mL DMF. After vigorous stirring, the vial was capped and heated in a thermostat at 85°C for 24 h. After the reaction is completed, it is naturally cooled to room temperature. The product was collected by centrifugation and washed with dichloromethane. It was then dried overnight in a freeze dryer for characterization.

Synthesis of ZIF-95. In a typical synthesis of pure ZIF-95, 237.5 mg $Zn(NO_3)_2·6H_2O$ and 1221 mg HcbIm were added to a beaker containing 55 ml DMF and 5 ml water. After stirring for 2 h, the mixed solution was added to a stainless steel autoclave lined with Teflon, and then heated in an air circulating oven at 120 °C for 3 days. The product was collected by centrifugation, washed with methanol and DMF, and then dried overnight in a freeze dryer for characterization.

Synthesis of N doped carbon from ZIF materials. The as-prepared ZIF-EC1, ZIF-1, ZIF-8 and ZIF-95 materials were annealed at 900 °C for 2 h at a ramp rate of 5 °C min^{-1} in flowing Ar in a tube furnace. Finally, the N doped carbon electrocatalysts were obtained.

Section 3. Structural analysis by cRED

The samples for high throughput cRED investigations were crushed in a mortar and dispersed in deionized water. A droplet was then taken from the suspension, transferred to a copper grid covered with lacey carbon, and dried in air at room temperature. cRED data were collected on a JEOL JEM2100 microscope operated at 200 kV (Cs 1.0 mm, point resolution 0.23 nm). TEM images were recorded with a Gatan Orius 833 CCD camera (resolution 2048 x 2048 pixels, pixel size 7.4 μm). cRED data collection was controlled by using the data acquisition software *Instamatic*[1], and electron diffraction (ED) frames were recorded with a Timepix hybrid detector QTPX-262k (512 x 512 pixels, pixel size 55 μm, max 120 frames/second, Amsterdam Sci. Ins.). A single-tilt holder was used for the data collection, which could tilt from –60° to +60° in the TEM. The area used for cRED data collection was about 1.0 μm in diameter, defined using a selected-area aperture. To minimize electron beam damage on the crystals, a low electron dose and high

rotation speed were applied. The high throughput cRED method can benefit for virtualization and identification of each individual nanocrystals. As shown in Figure 1b, ~30 nanocrystals can be analyzed in an area of 35 ×35 μm^2. By taking advantage of continuous rotation, it takes less than 5 minutes to collect a complete cRED dataset. For ZIF-CO_3-1, a typical cRED dataset covered a crystal rotation angle of 100.2° and took 3.7 min to collect. For ZIF-EC1, the rotation range was 117.5° and the collection time was 4.3 min.

The obtained cRED data were analyzed by using *REDprocessing* software package[2]. Two sets of unit cells and space groups were determined from 11 nanocrystals in phase mixture, indicating it contains two different structures. For the major phase, one typical unit cell was determined as a = 10.57 Å, b = 12.40 Å, c = 4.65 Å, α = 90.8°, β = 90.9°, and γ = 91.6°. The intensity distribution of reflections in the 3D reciprocal lattice indicates that the crystal is orthorhombic with a Laue class of *mmm* (Figures S2a-d). The unit cell angles α, β, and γ are near 90°, which also confirms the orthorhombic crystal system. The reflection conditions were deduced from the 2D slice cuts as $0kl$: $k = 2n$; $h0l$: $h = 2n$; $h00$, $h = 2n$; $0k0$: $k = 2n$, which corresponds to two possible space groups of *Pba*2 (No. 32), and *Pbam* (No. 55). For the minor phase, the unit cell parameters were determined to be a = 13.65 Å, b = 14.36 Å, c = 14.30 Å, α = 90.3°, β = 117.5°, and γ = 90.6°. The intensity distribution of reflections in the 3D reciprocal lattice indicates that the crystal is monoclinic with a Laue class of 2/*m* (Figures S2e-f). The reflection conditions was deduced as $0k0$: k=2n; $00l$: l=2n; $h0l$: l=2n, which corresponds to the space group: $P2_1/c$ (No. 14). The details of data collection and unit cell determination are summarized in Table S1.

After changing the synthetic conditions, we applied the high throughput cRED method on the product obtained using the optimized synthesis condition for ZIF-EC1. The sample purity was further confirmed by Pawley fitting of the PXRD pattern (Figure S6). With improved crystallinity, cRED dataset with higher resolution and higher completeness were obtained (Figure S7). The intensities of the reflections were extracted from the cRED data using the X-ray Detector Software (*XDS*)[3]. To obtain a high data completeness, nine cRED datasets were merged to a resolution of 0.78 Å and a completeness of 89.5% using *XSCALE*. Structure solution and refinement were conducted by using the SHELX software package[5]. In the refinement of the merged dataset, distance (DFIX) and planarity (FLAT) restraints were applied to the 2-methylimidazolate linkers to maintain a reasonable geometry. EADP was applied on two carbon atoms on the imidazolate group. In addition, EXTI was used in the final refinement, which converged with the agreement values R_1=0.1811 for 4103 $F_o > 4\sigma(F_o)$ and 0.1984 for all 5116 data for 302 parameters (Table S2).

After knowing the structure of ZIF-EC1, we calculated its metal and nitrogen density and compared them to most reported ZIFs (Table 1). The metal density was calculated as

$$metal\ density = \frac{number\ of\ metal\ atoms\ per\ unit\ cell}{unit\ cell\ volume}$$

where the number of metal atoms per unit cell and the unit cell volume are read from the cif file. For example, the Zn atom density in ZIF-EC1 was calculated as

$$\frac{12}{2514.7\ Å^3} = 4.77\ nm^{-3}$$

The nitrogen density is calculated in a similar way as

$$nitrogen\ density = \frac{number\ of\ nitrogen\ atoms\ per\ unit\ cell}{unit\ cell\ volume}$$

For example, the N atom density in ZIF-EC1 was calculated as

$$\frac{40}{2514.7\ Å^3} = 15.90\ nm^{-3}$$

Section 4. Energy calculation of ZIF-EC1

Atomic Simulation Environment (ASE)[4] was used to handle the simulation and the QUANTUM ESPRESSO program package[5] to perform electronic structure calculations. The electronic wavefunctions were expanded in plane waves up to a cutoff energy of 800 eV, while the electron density is represented on a grid with an energy cutoff of 8000 eV after carrying out the convergence tests. Core electrons were approximated using ultrasoft pseudopotentials[6]. The ground state energies of ZIF-EC1, ZIF-CO_3-1 and ZIF-8 bulk structures were calculated using PBE exchange-correlation functional with dispersion correction[7]. The Brillouin zone were converged and sampled with (3 × 3 × 3) Monkhorst-Pack k-points.

Section 5. Electrochemical analysis of ZIF-EC1 and its derivatives

The potentials corresponding to the reversible hydrogen electrode (RHE) electrode were calculated with the following equation:

$$E_{RHE} = E_{Ag/AgCl} + (0.197 + 0.0591 \times \mathrm{pH}) \qquad Eq\ (1)$$

The electron transfer numbers (n) were calculated with Koutecky-Levich (K-L) equation:

$$1/j = 1/j_l + 1/j_k = 1/B\omega^{1/2}/ + 1/j_k \qquad Eq\ (2)$$

where j is the measured current density; j_l is the diffusion current density; j_k is the kinetic current density; ω is the rotation speed in rpm; B can be confirmed by Koutecky-Levich (K-L) equation:

$$\mathrm{B} = 0.2\mathrm{nFC_0}(D_0)^{2/3}/\upsilon^{-1/6} \qquad Eq\ (3)$$

where n is the transfer number; F is the Faraday constant (96485 C mol^{-1}); C_0 is the concentration of O_2 in 0.1 M KOH (1.2×10^{-6} mol cm^{-3}); D_0 is the diffusion coefficient of O_2 in 0.1 M KOH (1.9×10^{-5} cm^2 s^{-1}); υ is the viscosity of 0.1 M KOH (0.1 cm^2 s^{-1}). The transfer number (*n*) was obtained by using the equations.

The LSV curve of ZIF-EC1 in 0.1 M KOH solution saturated by O_2 is shown in Figure S9. The $E_{1/2}$ of ZIF-EC1 was measured to be 0.757 V, indicating a moderate ORR activity. The high density of surface Zn-N_x structure could attribute to the good ORR activity. However, MOF materials are limited by their conductivities when being used as electrocatalysts for ORR. ZIF-EC1 possesses a high density of activity sites. NC-ZIF-EC1 derived from ZIF-EC1 has the highest N loading compared to those of NC-ZIF-1, NC-ZIF-8, and NC-ZIF-95 (Figures S15, S16 and Table S4). The I_D/I_G of NC-ZIF-EC1, NC-ZIF-1, NC-ZIF-8, and NC-ZIF-95 is 1.08, 1.35, 1.04, 1.08, respectively (Figure S17). PXRD shows that all samples exhibit two similar broad peaks assigned to partial graphitized carbon. The results shown in Figure 5 indicates that NC-ZIF-EC1 exhibits the best ORR activity among all carbon based materials derived from ZIFs, which is also compatible to that of commercial Pt/C. A Tafel slope of 96.9 mV dec^{-1} is observed for NC-ZIF-EC1, which is considerably lower than that of NC-ZIF-1 (135.5 mV dec^{-1}), NC-ZIF-8 (103.4 mV dec^{-1}), and NC-ZIF-95 (215.8 mV dec^{-1}) (Figure 5c). This indicates a favorable reaction kinetics for NC-ZIF-EC1 compared to NC-ZIF-1, NC-ZIF-8, and NC-ZIF-95.

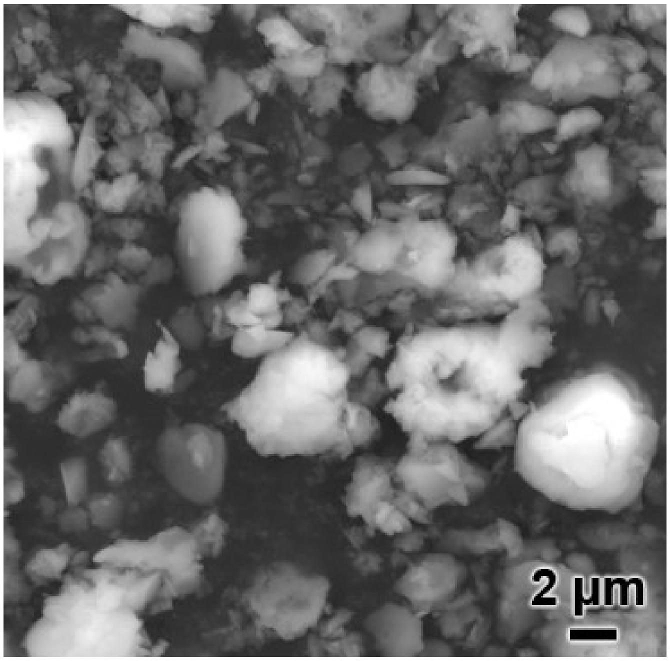

Figure S1. SEM image of the obtained product, showing a variation of particle sizes and morphologies.

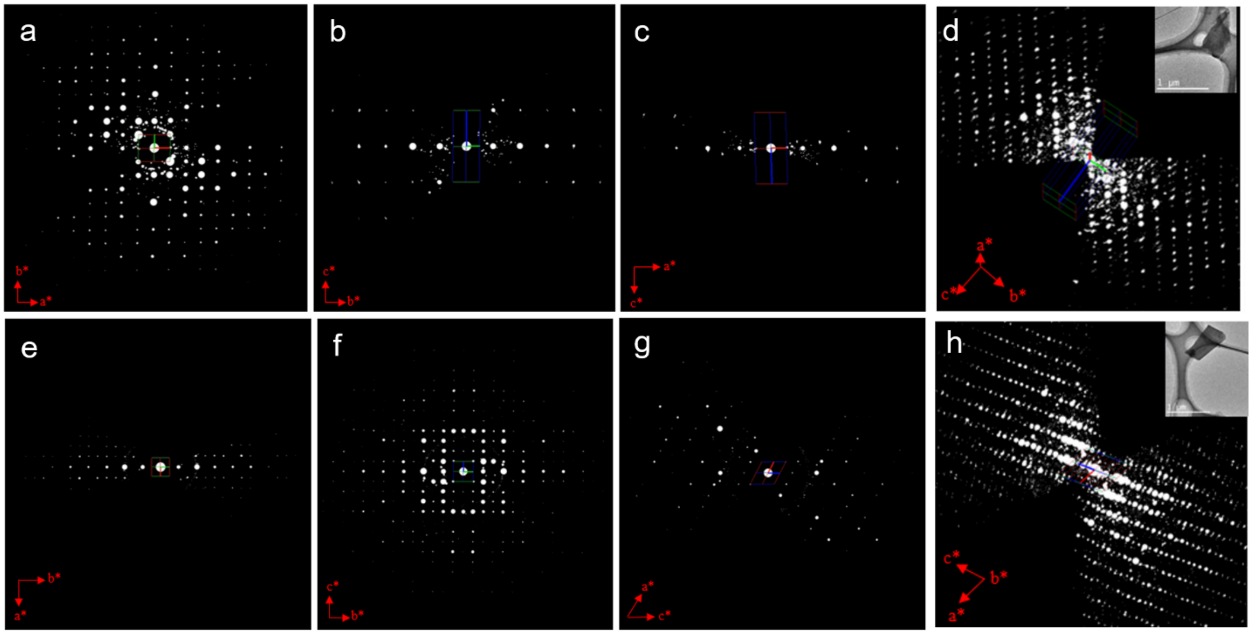

Figure S2. (a-c) 2D slice cuts from the reconstructed 3D reciprocal lattice of ZIF-CO_3-1 showing the (a) *hk*0, (b) 0*kl*, and (c) *h*0*l* planes. (d) 3D reciprocal lattice of ZIF-CO_3-1. (e-g) 2D slice cuts from the reconstructed 3D reciprocal lattice of ZIF-EC1 showing the (e) *hk*0, (f) 0*kl*, and (g) *h*0*l* planes. (h) 3D reciprocal lattice of ZIF-EC1.

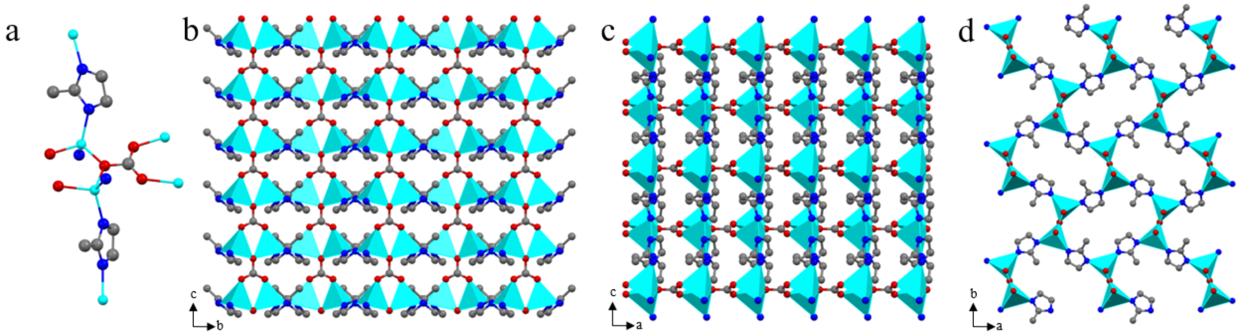

Figure S3. Structural model of ZIF-CO_3-1. (a) The coordination geometry of Zn. (b-d) The framework structure viewing along *a*-, *b*- and *c*-axis, respectively. Cyan tetrahedra: Zn atoms; red spheres: O atoms; blue spheres: N atoms; grey spheres: C atoms.

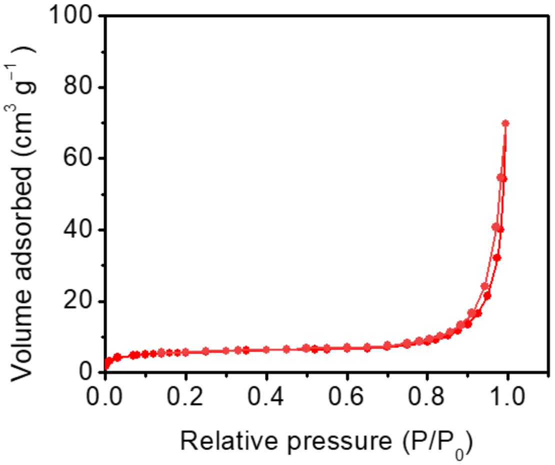

Figure S4. N_2 adsorption-desorption isotherm of ZIF-EC1. ZIF-EC1 is nonporous to N_2. The porosity showed in the P/P$_o$ range > 0.9 is caused by interparticle voids. The BET surface area is 19.7 m^2 g^{-1}.

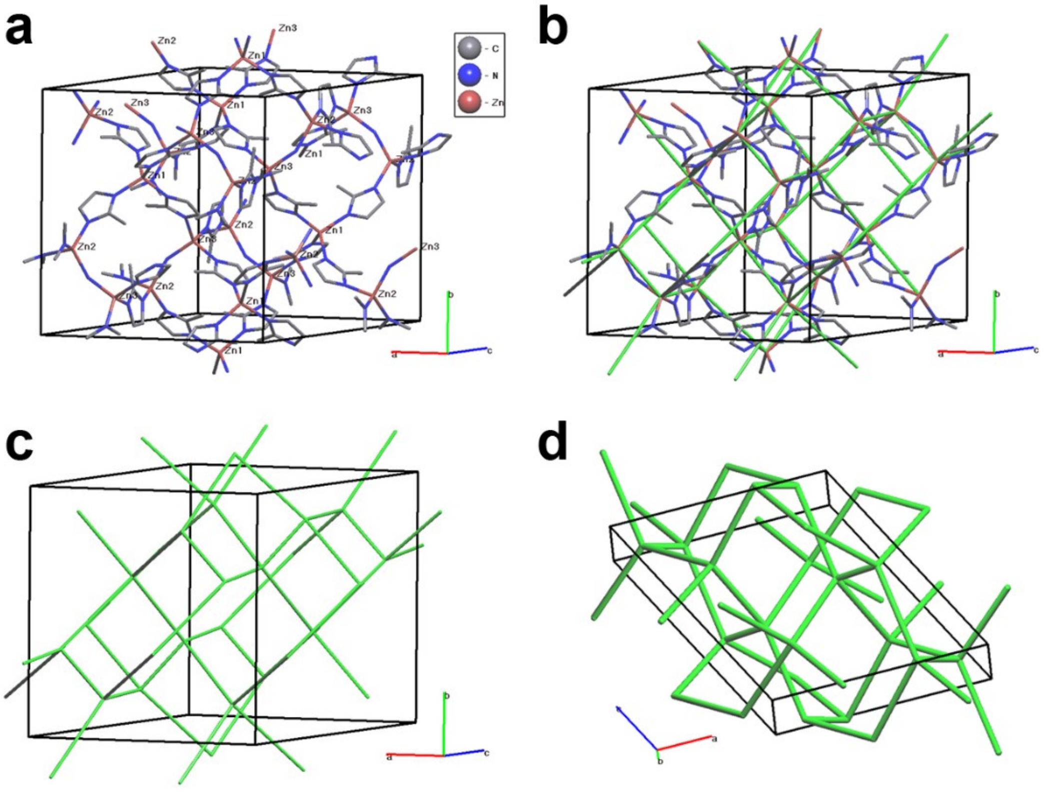

Figure S5. (a) The structural model of ZIF-EC1. (b-d) The definition of the underlying net, which is showin in green. Each of the three independent Zn are 4-c nodes and each ligand is bridging two Zn atoms. From the coordinates of the underlying net, ToposPro find that is a known binodal net (meaning that two of the three Zn atoms are topologically equivalent) called *yqt*1. It is defined as a 4,4-c net with stoichiometry (4-c)(4-c)2, and a 2-nodal net with the point symbol of $(5.6^2.7^2.8)(5^2.6.7.8^2)_2$, and the vertex symbol of $[5.8_4.5.8_5.6.8_5]$ $[5.8_5.6.6.8_4.8_4]$.

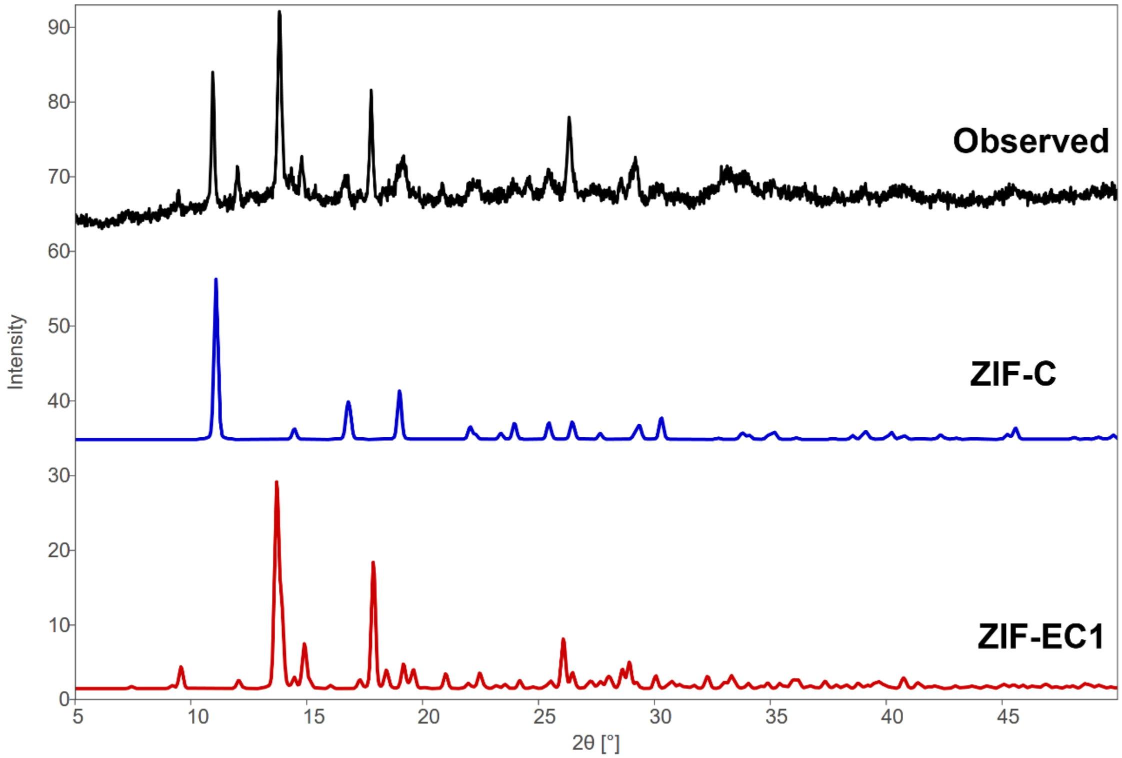

Figure S6. Comparison of the observed PXRD pattern of the phase mixture with the simulated PXRD patterns of ZIF-CO_3-1 and ZIF-EC1. All peaks in the observed pattern can be indexed using the two phases. The PXRD patterns were simulated from the corresponding structural models using a pseudo-Voigt peak shape function.

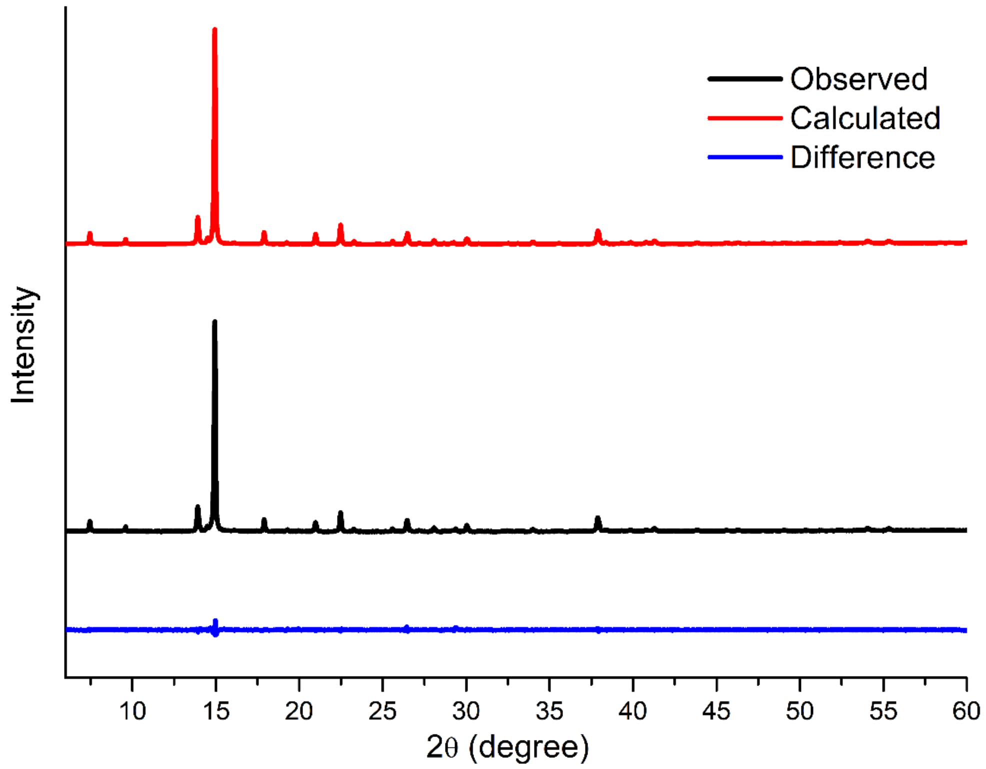

Figure S7. Pawley fit of the experimental PXRD pattern (λ = 1.5406 Å) of ZIF-EC1, which shows a good agreement indicating the sample is pure ZIF-EC1.

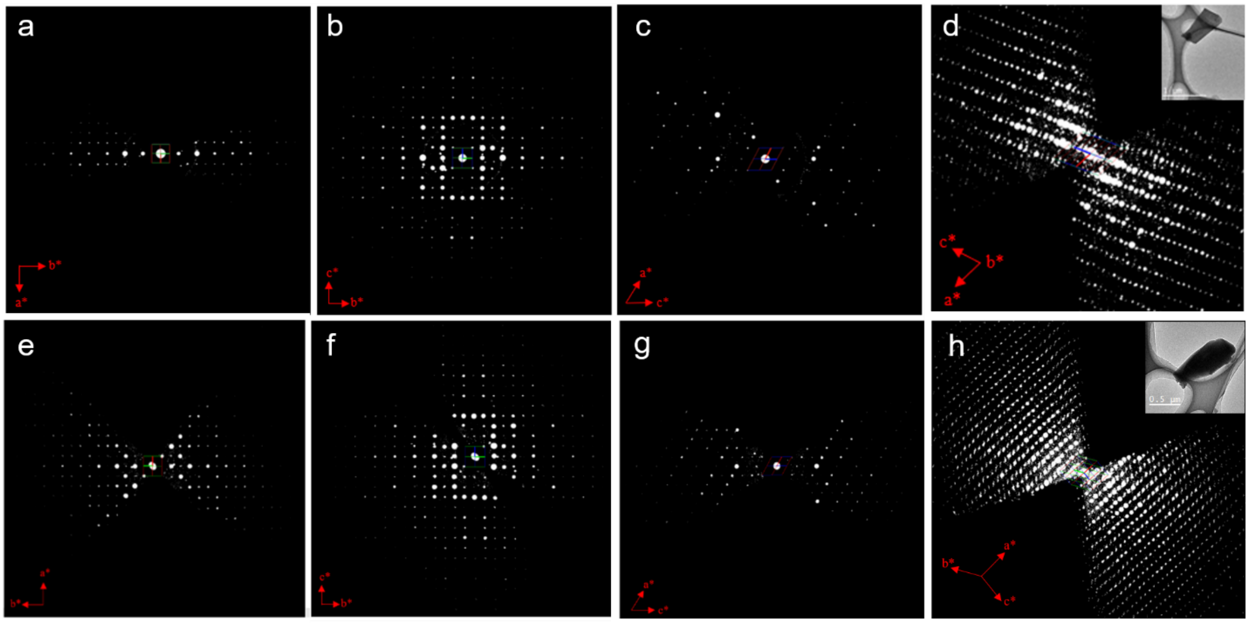

Figure S8. Reciprocal lattices reconstructed from cRED data. (a-d) 2D slice cuts of (a) *hk*0, (b) 0*kl*, and (c) *h*0*l* planes from the reconstructed 3D reciprocal lattice (d) of a ZIF-EC1 crystal (inset in d) in the phase mixture. (e-h) 2D slice cuts of (e) *hk*0, (f) 0*kl*, and (g) *h*0*l* planes from the reconstructed 3D reciprocal lattice (h) of a ZIF-EC1 crystal (inset in h) in the pure ZIF-EC1 sample.

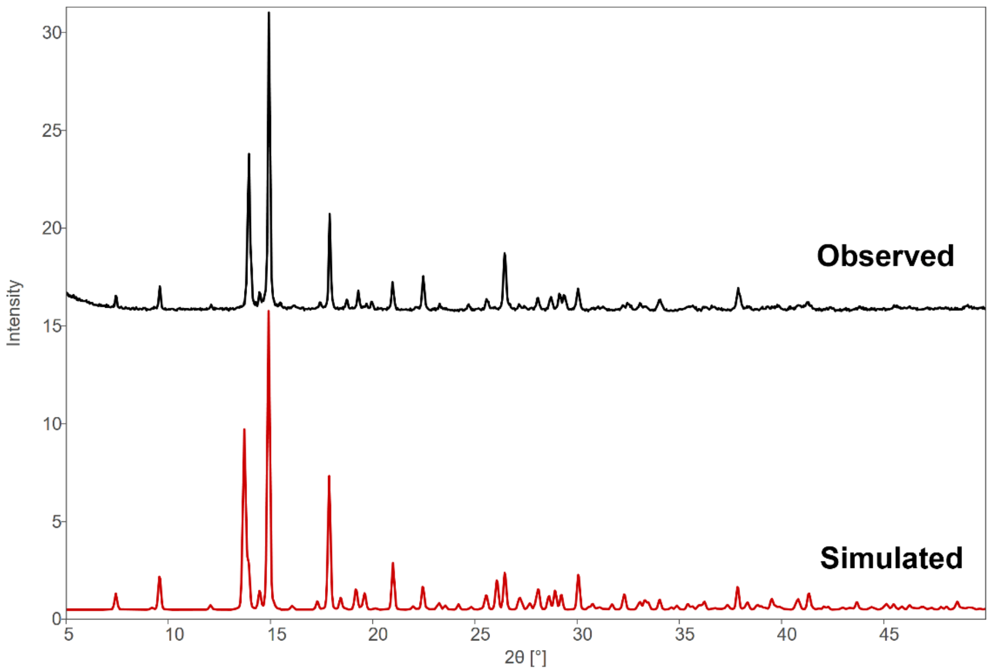

Figure S9. Comparison of observed and simulated PXRD patterns of ZIF-EC1 (λ = 1.5406 Å). The PXRD pattern were simulated using a pseudo-Voigt peak shape function, with the preferred orientation simulated using a weighted March-type correction.

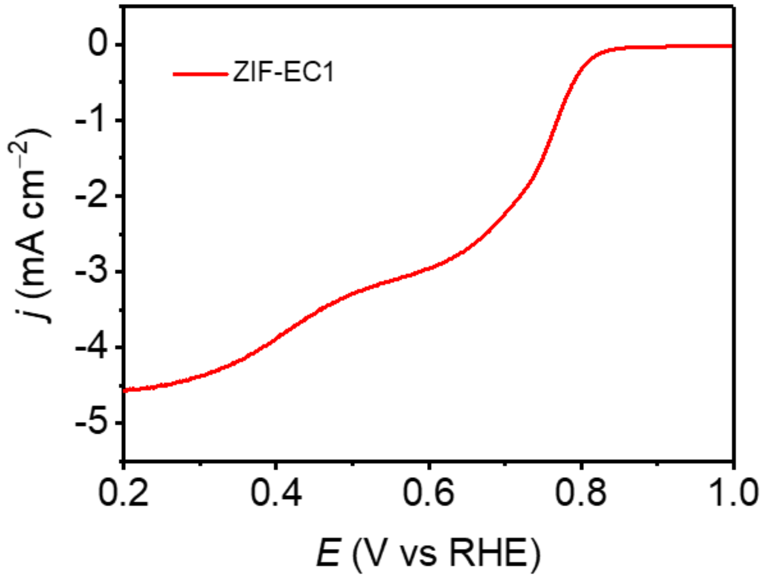

Figure S10. LSV curves of ZIF-EC1 in 0.1 M KOH solution saturated by O_2. The ZIF-EC1 sample was mixed with 50% carbon black. The $E_{1/2}$ of ZIF-EC1 was measured to be 0.757 V, indicating a moderate ORR activity.

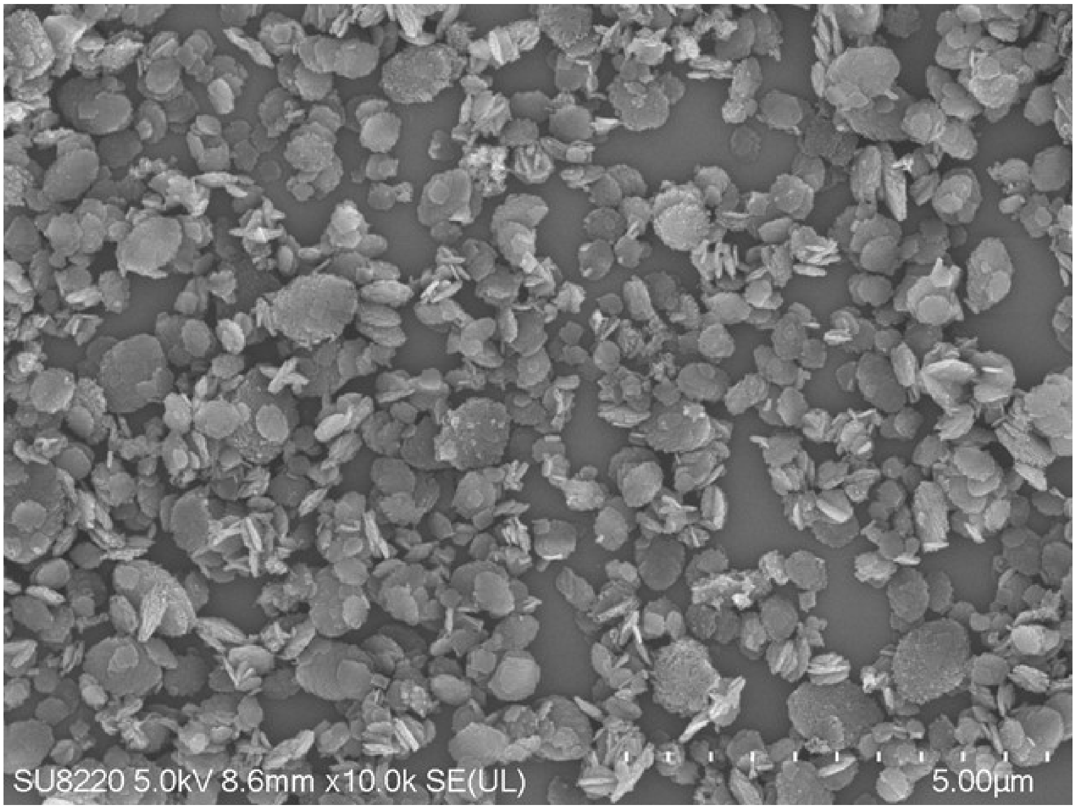

Figure S11. SEM image of NC-ZIF-EC1 nanocrystals.

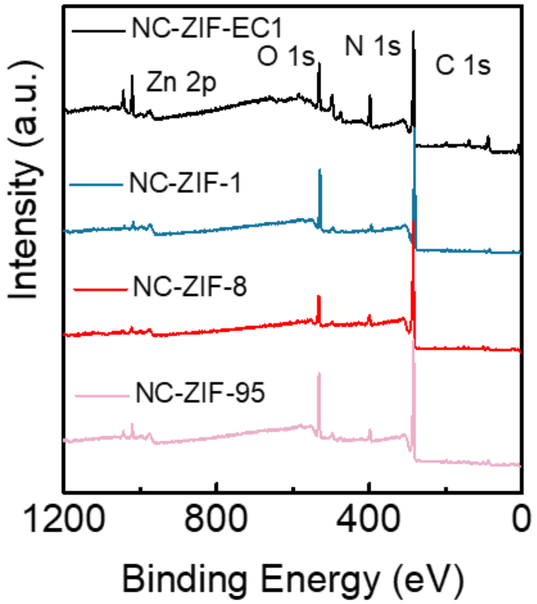

Figure S12. XPS spectra of NC-ZIF-EC1, NC-ZIF-1, NC-ZIF-8, and NC-ZIF-95. The contents of C, N, O, Zn calculated based on XPS are summarized in Table S4.

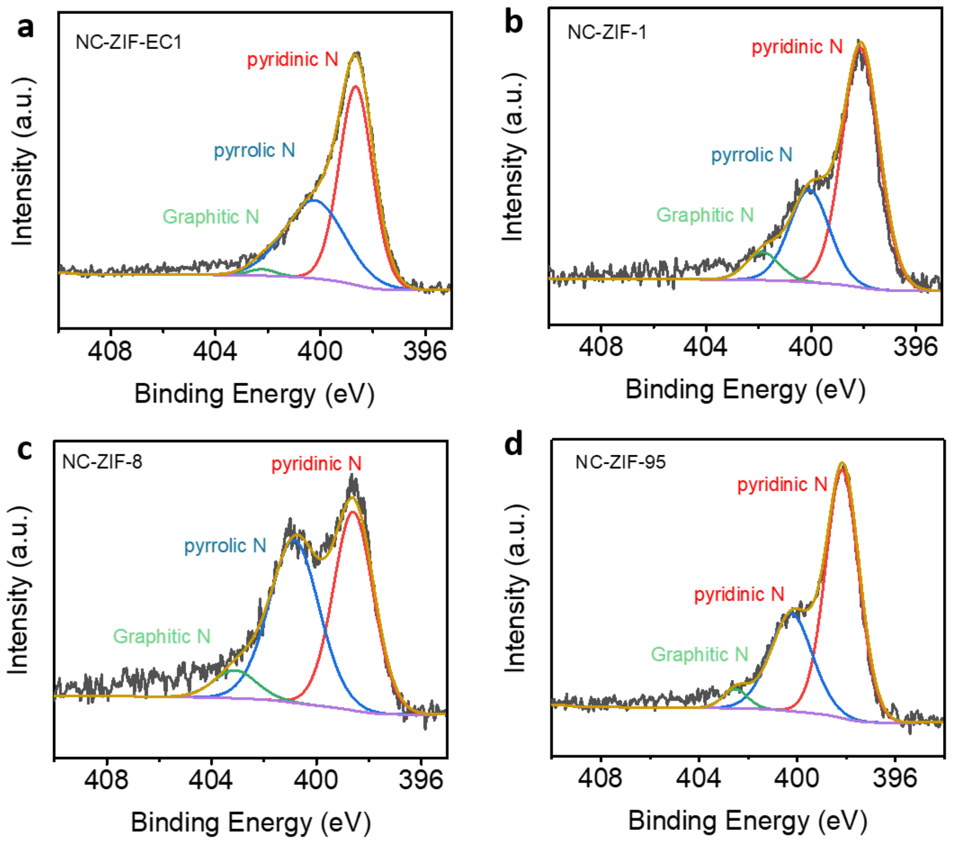

Figure S13. High-resolution XPS spectra of N 1s for NC-ZIF-EC1 (a), NC-ZIF-1 (b), NC-ZIF-8 (c), and NC-ZIF-95 (d).

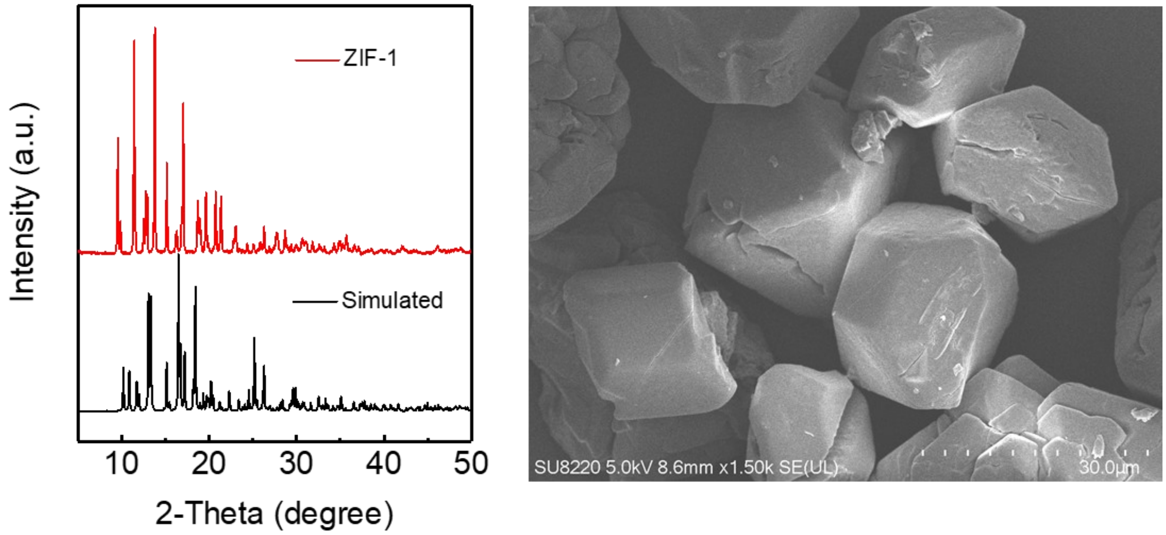

Figure S14. PXRD pattern (left) and SEM image (right) of ZIF-1.

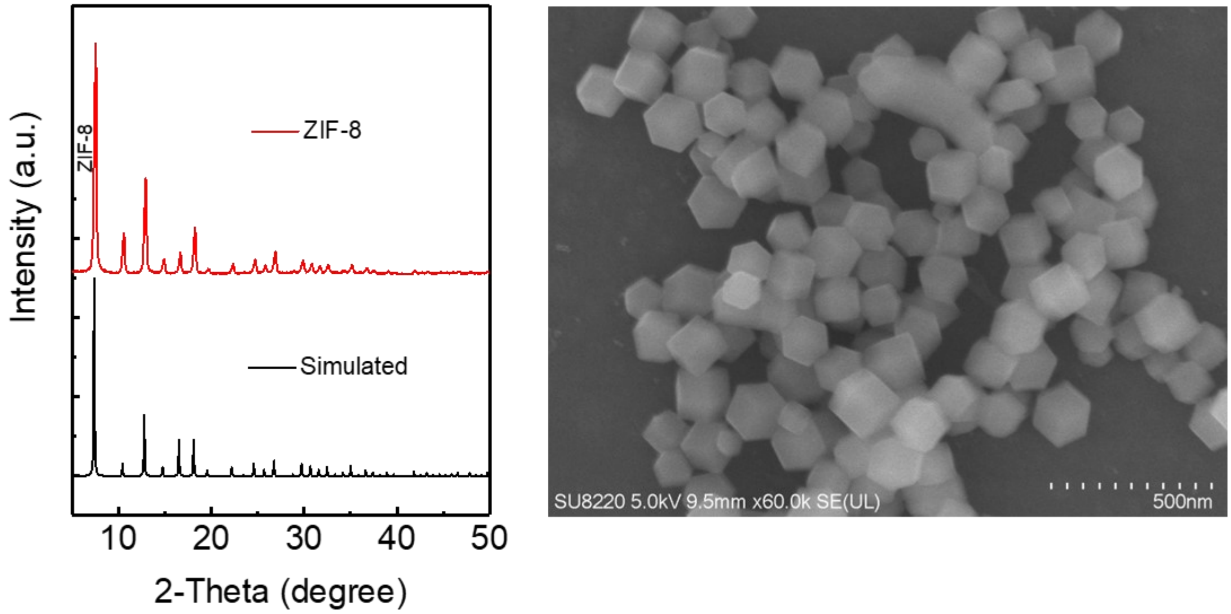

Figure S15. PXRD pattern (left) and SEM image (right) of ZIF-8.

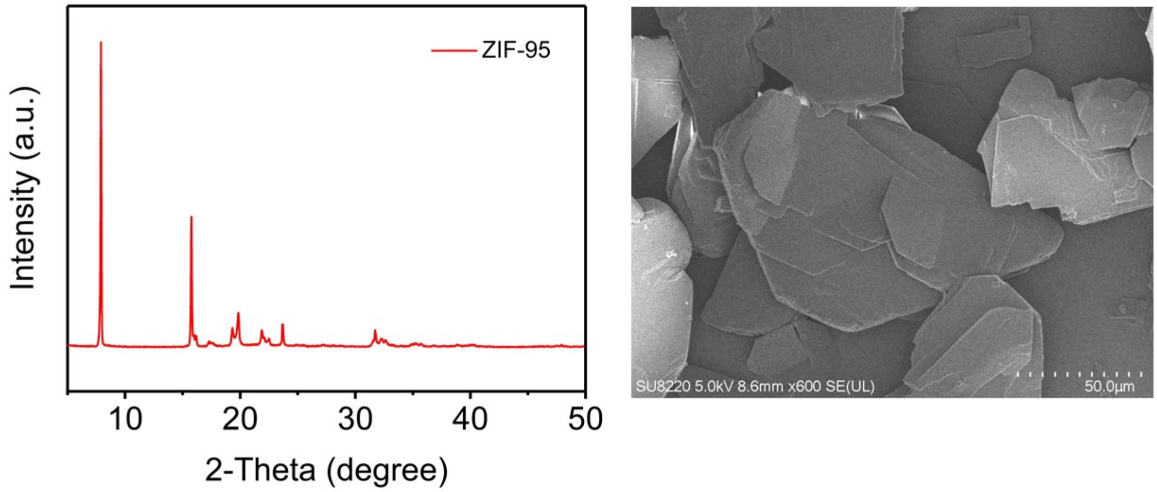

Figure S16. PXRD pattern (left) and SEM image (right) of ZIF-95.

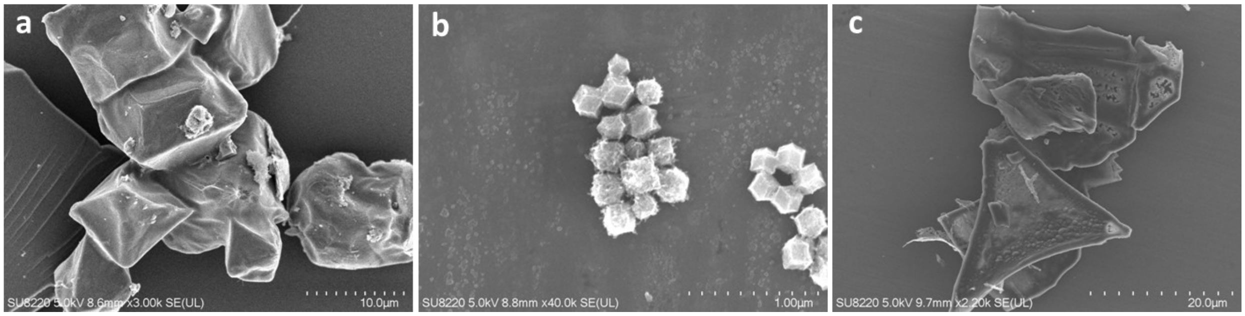

Figure S17. SEM images of (a) NC-ZIF-1, (b) NC-ZIF-8, and (c) NC-ZIF-95.

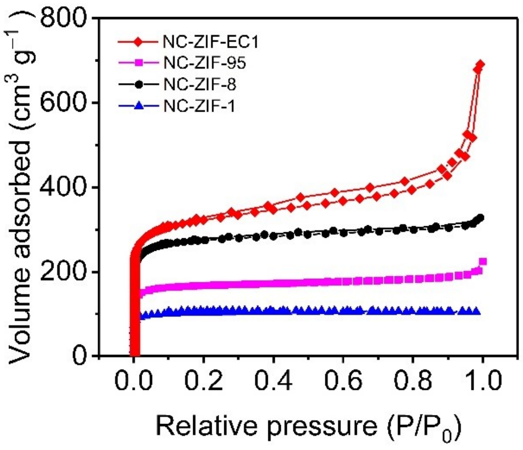

Figure S18. N_2 adsorption-desorption isotherm of NC-ZIF-EC1, NC-ZIF-1, NC-ZIF-8, and NC-ZIF-95, from which the Brunauer-Emmett-Teller (BET) surface areas are estimated as 1226, 1079, 657, and 295 m^2g^{-1}, respectively.

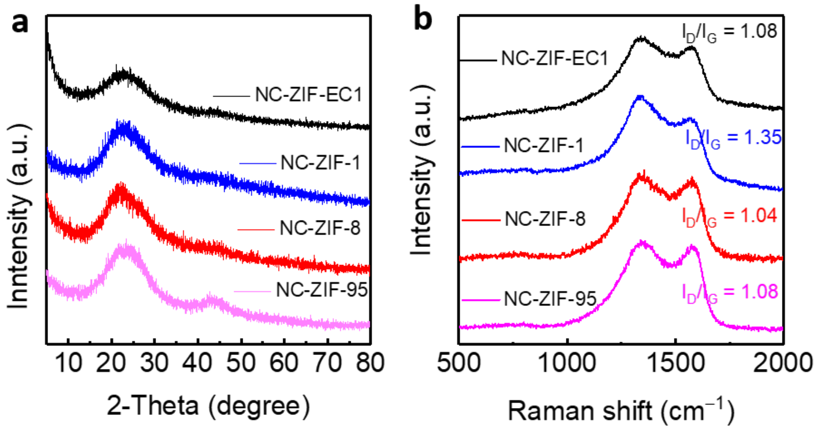

Figure S19. PXRD patterns (a) and Raman spectra (b) of various N doped carbon materials derived from different ZIFs.

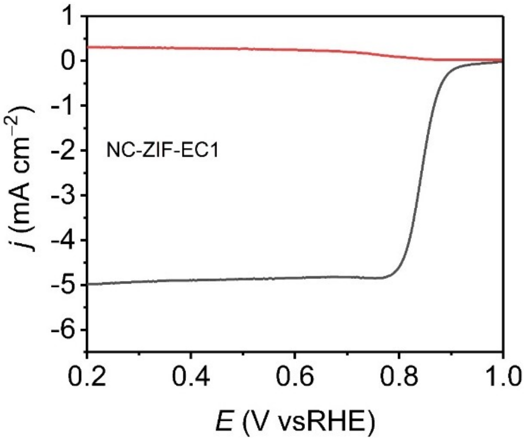

Figure S20. LSV curves of the NC-ZIF-EC1 in O_2-saturated 0.1 M KOH solution using a rotating ring disk electrode with a scan rate of 5 mV s^{-1} and a rotation speed of 1600 rpm.

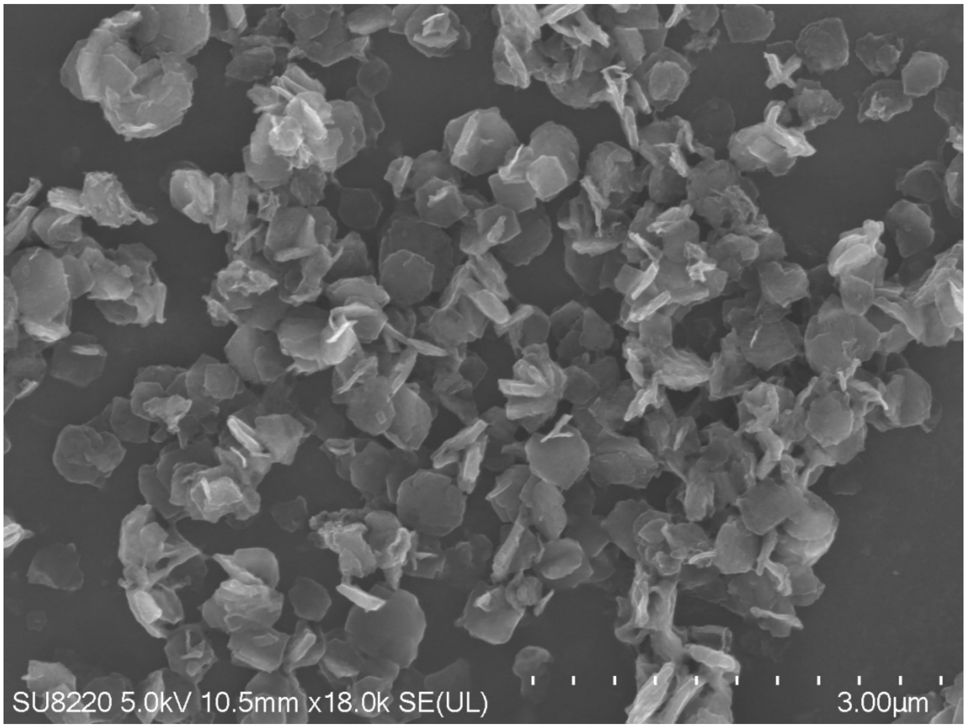

Figure S21. SEM image of NC-ZIF-EC1 after ORR reaction.

Table S1. Details of cRED data collection on different nanocrystals in the phase mixture, and the corresponding unit cell parameters and space groups deduced from the cRED data.

Dataset	1	2	3	4	5	6	7	8	9	10	11
Phase	ZIF-CO_3-1									ZIF-EC1	
Rotation range (°)	100.18	103.57	93.91	60.31	74.01	54.06	48.35	43.60	39.26	117.45	42.34
Rotation step (°)	0.9	0.9	0.9	0.9	0.9	0.9	0.9	0.9	0.9	1.5	0.9
Exposure time (s $frame^{-1}$)	0.5	0.5	0.5	0.5	0.5	0.5	0.5	0.5	0.5	0.3	0.5
No. of frames	390	402	365	238	287	208	187	170	153	748	165
Total data collection time (min)	3.7	3.8	3.5	2.3	2.7	2.0	1.8	1.6	1.5	4.3	1.6
Crystal system	Orthorhombic									Monoclinic	
Space group	*Pba*2 (No. 32) or *Pbam* (No. 55)									$P2_1/c$	
a (Å)	10.501	10.500	10.547	10.474	10.513	10.89	10.94	10.84	10.81	13.579	14.035
b (Å)	12.507	12.400	12.236	12.162	12.331	12.84	12.63	12.52	12.93	14.550	15.379
c (Å)	4.691	4.600	4.669	4.548	4.664	4.88	4.77	4.68	4.87	14.314	14.620
α (°)	90	90	90	90	90	90	90	90	90	90.000	90.000
β (°)	90	90	90	90	90	90	90	90	90	117.98	118.419
γ (°)	90	90	90	90	90	90	90	90	90	90.000	90.000

Table S2. Crystallographic data and refinement details of ZIF-EC1.

	Phase mixture	Pure phase*
Wavelength (Å)	0.02508 Å	0.02508 Å
Resolution (Å)	0.90	0.78
Crystal system	Monoclinic	Monoclinic
Space group	$P2_1/c$ (No. 14)	$P2_1/c$ (No. 14)
a (Å)	13.579(3)	13.462(2)
b (Å)	14.550(3)	14.659(3)
c (Å)	14.314(3)	14.449(2)
β (°)	117.98(3)	118.12(1)
Volume (Å^3)	2497.5(11)	2514.7(8)
Completeness (%)	61.4	89.5
No. unique reflections	2008	5116
No. observed reflections (I > 2 sigma(I))	878	4103
R_1 (I > 2 sigma(I))	0.238	0.181
R_1 (all reflections)	0.301	0.198
Goof	1.314	1.725

* Nine datasets are merged.

Table S3. Crystallographic data and Pawley fitting results of ZIF-EC1.

Chemical formula	$C_{20}N_{10}OZn_3$
Formula weight	592.47
Crystal system	Monoclinic
Space group	$P2_1/c$ (No. 14)
a/Å	13.461(2)
b/Å	14.659(3)
c/Å	14.449(2)
β/°	118.12(2)
Temperature/K	298(2)
Wavelength/Å	1.54056
2θ range/°	5.998356 – 59.990356
R_p	0.0471
R_{wp}	0.0667
R_{exp}	0.0429
GOOF	1.552

Table S4. Elemental contents (wt%) in different catalysts estimated by XPS.

Catalyst	C	N	O	Zn
NC-ZIF-EC1	69.49±0.016	13.41±0.02	12.95±0.03	2.12±0.71
NC-ZIF-1	77.02±0.021	4.05±0.011	17.11±0.092	0.49±0.077
NC-ZIF-8	80.74±0.013	6.52±0.054	10.02±0.081	0.32±0.12
NC-ZIF-95	75.23±0.034	6.42±0.143	16.46±0.094	0.71±0.044

Table S5. Comparison of the onset potential E_{onset} and half-wave potential $E_{1/2}$ of NC materials for ORR.

Catalyst	E_{onset} vs RHE (V)	$E_{1/2}$ vs RHE (V)
NC-ZIF-EC1	0.930	0.860
NC-ZIF-1	0.880	0.807
NC-ZIF-8	0.910	0.836
NC-ZIF-95	0.855	0.720
Pt/C	0.940	0.867

Table S6. Comparison of the performance of NC-ZIF-EC1 with previously reported electrocatalysts.

Catalyst	Electrolyte	$E_{1/2}$ (vs RHE)	Ref.
**NC-ZIF-EC1**	**0.1 M KOH**	**0.860 V**	**This work**
Zn-N-C	0.1 M KOH	0.850V	[8]
ZnN_x/BP	0.1 M KOH	0.837 V	[9]
Zn-NC-1	0.1 M KOH	0.873 V	[10]
CoZn-NC-700	0.1 M KOH	0.840 V	[11]
CoFeZn/NC	0.1 M KOH	0.850 V	[12]
M (M = Co and Zn)$_2P_2O_7$@NC	0.1 M KOH	0.793 V	[13]
ZCP-CFs-9 (Zn, Co-ZIF)	0.1 M KOH	0.829 V	[14]
$Ni_3(HITP)_2$	0.1 M KOH	0.82 V	[15]
Fe-TAPP COF aerogel	0.1 M KOH	0.83 V	[16]
2D CAN-Pc(Fe/Co)	0.1 M KOH	0.84 V	[17]
PcCu-O_8-Co MOF	0.1 M KOH	0.83 V	[18]
NCo@CNT-NF700	0.1 M KOH	0.87 V	[19]
MnO/Co/PGC	0.1 M KOH	0.78 V	[20]
Co_2VO_4	1.0 M KOH	0.83 V	[21]

NC=Nitrogen doped carbon; BP=carbon black; CF=carbon fibers; HITP=hexaiminotriphenylene; TAPP=5,10,15,20-(Tetra-4-aminophenyl)porphyrin; CAN=conjugated aromatic networks; Pc=phthalocyanine; CNT=carbon nanotube; NF=nanofiber; PGC=porous graphitic carbon

References

[1] M. O. Cichocka, J. Ångström, B. Wang, X. Zou, S. Smeets, *J. Appl. Crystallogr.* **2018**, *51*, 1652–1661.
[2] W. Wan, J. Sun, J. Su, S. Hovmöller, X. Zou, *J. Appl. Crystallogr.* **2013**, *46*, 1863–1873.
[3] W. Kabsch, *Acta Crystallogr. D Biol. Crystallogr.* **2010**, *66*, 133–144.
[4] S. R. Bahn, K. W. Jacobsen, *Comput. Sci. Eng.* **2002**, *4*, 56–66.
[5] P. Giannozzi, S. Baroni, N. Bonini, M. Calandra, R. Car, C. Cavazzoni, D. Ceresoli, G. L. Chiarotti, M. Cococcioni, I. Dabo, A. D. Corso, S. de Gironcoli, S. Fabris, G. Fratesi, R. Gebauer, U. Gerstmann, C.

Gougoussis, A. Kokalj, M. Lazzeri, L. Martin-Samos, N. Marzari, F. Mauri, R. Mazzarello, S. Paolini, A. Pasquarello, L. Paulatto, C. Sbraccia, S. Scandolo, G. Sclauzero, A. P. Seitsonen, A. Smogunov, P. Umari, R. M. Wentzcovitch, *J. Phys. Condens. Matter* **2009**, *21*, 395502.
[6] A. A. Adllan, A. D. Corso, *J. Phys. Condens. Matter* **2011**, *23*, 425501.
[7] S. Grimme, J. Antony, S. Ehrlich, H. Krieg, *J. Chem. Phys.* **2010**, *132*, 154104.
[8] J. Wang, H. Li, S. Liu, Y. Hu, J. Zhang, M. Xia, Y. Hou, J. Tse, J. Zhang, Y. Zhao, *Angew. Chem. Int. Ed.* DOI: 10.1002/anie.202009991.
[9] P. Song, M. Luo, X. Liu, W. Xing, W. Xu, Z. Jiang, L. Gu, *Adv. Funct. Mater.* **2017**, *27*, 1700802.
[10] J. Li, S. Chen, N. Yang, M. Deng, S. Ibraheem, J. Deng, J. Li, L. Li, Z. Wei, *Angew. Chem. Int. Ed.* **2019**, *58*, 7035–7039.
[11] B. Chen, X. He, F. Yin, H. Wang, D.-J. Liu, R. Shi, J. Chen, H. Yin, *Adv. Funct. Mater.* **2017**, *27*, 1700795.
[12] J. Wang, W. Zang, S. Xi, M. Kosari, S. J. Pennycook, H. Chun Zeng, *J. Mater. Chem. A* **2020**, *8*, 17266–17275.
[13] L.-H. Xu, H.-B. Zeng, X.-J. Zhang, S. Cosnier, R. S. Marks, D. Shan, *J. Catal.* **2019**, *377*, 20–27.
[14] C. Liu, J. Wang, J. Li, J. Liu, C. Wang, X. Sun, J. Shen, W. Han, L. Wang, *J. Mater. Chem. A* **2017**, *5*, 1211–1220.
[15] E. M. Miner, T. Fukushima, D. Sheberla, L. Sun, Y. Surendranath, M. Dincă, *Nat. Commun.* **2016**, *7*, 10942.
[16] N. Zion, D. A. Cullen, P. Zelenay, L. Elbaz, *Angew. Chem. Int. Ed.* **2020**, *59*, 2483–2489.
[17] S. Yang, Y. Yu, M. Dou, Z. Zhang, L. Dai, F. Wang, *Angew. Chem. Int. Ed.* **2019**, *58*, 14724–14730.
[18] H. Zhong, K. H. Ly, M. Wang, Y. Krupskaya, X. Han, J. Zhang, J. Zhang, V. Kataev, B. Büchner, I. M. Weidinger, S. Kaskel, P. Liu, M. Chen, R. Dong, X. Feng, *Angew. Chem. Int. Ed.* **2019**, *58*, 10677–10682.
[19] L. Zou, C.-C. Hou, Z. Liu, H. Pang, Q. Xu, *J. Am. Chem. Soc.* **2018**, *140*, 15393–15401.
[20] X. F. Lu, Y. Chen, S. Wang, S. Gao, X. W. (David) Lou, *Adv. Mater.* **2019**, *31*, 1902339.
[21] C. Mu, J. Mao, J. Guo, Q. Guo, Z. Li, W. Qin, Z. Hu, K. Davey, T. Ling, S.-Z. Qiao, *Adv. Mater.* **2020**, *32*, 1907168.
